# The Prolongation of Life

**Published:** 1908-03

**Authors:** 


**The Prolongation of Life.** By ÉLIE METCHNIKOFF. The English translation edited by P. CHALMERS MITCHELL, M.A., D.Sc. (Oxon.), F.R.S. Pp. xx., 343. London: William Heinemann. 1907.

The title of this fascinating, brilliant, but not altogether convincing book does not indicate its full scope, but serves rather as a sort of link whereby its different sections are more or less loosely united. It is termed by its author a sequel to *The Nature*

*of Man,*[1] and from its pages may be obtained a detailed presentment of Professor Metchnikoff's point of view as regards human life as a whole, as well as his particular recommendations for prolonging its duration. The book is therefore of great interest and importance, and for this reason it is hoped that a review of rather greater length than is usual in the *Bristol Medico-Chirurgical Journal* will not be found unacceptable.

The general scheme of the work is somewhat as follows. Of the nine parts into which the book is divided, the first four deal explicitly with the duration of life. The first part is entitled "The Investigation of Old Age;" its object is to show that senility is caused by the activity of certain phagocytic cells, whereby the higher and more differentiated tissues, such as those of the central nervous system, are gradually absorbed and replaced by fibrous tissue. The part played by the internal secretions of such organs as the thyroid and the suprarenal capsules is considered as certainly secondary and possibly unimportant. The detailed observations on which Professor Metchnikoff founds this view are in the highest degree interesting and suggestive, and constitute by far the strongest part of the work, taken as a whole.

The second part deals with "Longevity in the Animal Kingdom," and is equally interesting, if not quite so convincing. Briefly, the author's thesis is that the duration of life in vertebrate animals varies inversely with the length of the colon. This is illustrated and supported by a reference to numerous animal types, and is explained by the assumption that senility and death are due to auto-intoxication from the products of putrefaction in the large intestine. Unfortunately for this theory, which is ingenious in conception and comprehensively supported by observation, the most notable exception happens to be that which interests us most, namely man himself. The long colon is here associated with a duration of life which, though not approaching that of the tortoise, is yet of very respectable proportions as compared to that of other mammals. How far such an important variant can be considered to nullify the general proposition is, of course, a difficult question; but in dealing with a topic which, after all, is highly theoretical, it certainly seems to us especially necessary to include satisfactorily all known facts.

The third part, "Investigations on Natural Death," appears to be of the nature of an intercalation, and in a certain sense to interrupt the sequence of thought. Possibly, however, it could not have been inserted elsewhere with any less inconvenience. Considered as a separate essay, it is of much interest, dealing as it does with the mode of death in many different kinds of beings. Natural death is defined as a death not due to external or

[1] Heinemann.

accidental causes, not dependent on inanition or the exhaustion consequent on such functions as reproduction, but a general atrophy of the tissues consequent on auto-intoxication. Professor Metchnikoff draws a somewhat elaborate parallel between "natural" death and sleep. The latter he inclines to regard as a temporary auto-intoxication; but in reality the experimental evidence for much of this theorising is, as Professor Halliburton has said, of the "slimsiest" character.

In Part IV., the question is propounded: "Should we try to prolong human life?" and, incidentally, the methods by which this may be compassed are sketched. After a review of the historical aspect of the subject, which shows a masterly power of condensing much erudition into a few short paragraphs without loss of lucidity, the author quotes Sir Hermann Weber's well-known address to the Royal College of Physicians, which was delivered in 1903. With the general advance in hygiene has come a corresponding lengthening of the average duration of life, and this is fully recognised by Professor Metchnikoff, and by other authorities, such as Sir Lauder Brunton, who have written on the subject. The Professor, however, is ready with three proposals for further advance in this direction. The first is the injection of a cytotoxic serum, which, though poisonous in large doses, would in minute amounts tend to strengthen the formed elements of the body, and so render them more resistant to those phagocytic processes which are the immediate cause of senile degeneration. Secondly, he is prepared with suggestions as to the large intestine. He points out the possibility of prolonged life after complete exclusion of considerable portions of the gut from functional activity. He had not, it appears, then been made acquainted with Mr. Arbuthnot Lane's work at Guy's Hospital, but we believe that emissaries from the Pasteur Institute have recently examined some of the cases on which these extensive operations have been successfully performed. For technical and other reasons, however, Metchnikoff is not quite prepared to advocate either of these methods; and seeing how difficult intestinal antisepsis appears to be in practice, he falls back on the somewhat humdrum expedient of advising prolonged mastication of food, in order that partially digested masses should not lie and putrify in the intestinal canal. However, as Dr. Hutchison has pointed out, the residue of food which escapes absorption under ordinary circumstances is only a small fraction of the whole, so that prolonged mastication, even if it led to complete absorption, could not materially affect the amount of toxic material in the colon. Whether, as Sir Lauder Brunton has suggested, undigested food masses may predispose to cancer by irritating the mucous membrane at such points as the pylorus is another question, into which the author does not enter.

Professor Metchnikoff then describes at some length his views on the advantage of eating cultures of the *Bacillus acidi lactici* as a check to intestinal putrefaction. The evidence he adduces is of a striking character, and consists partly of an account of experimental researches and partly of a description of numerous races of men who live largely on sour milk, and, according to the author, are notably long-lived. Whether or no longevity may be assured by eating the *Bacillus acidi lactici*, there seems very little doubt that it checks the growth of various other organisms which infest the intestinal canal in man. Soured milk has been successfully employed in diarrhœal conditions of infants, although some authors consider that excessive absorption of lactic acid gives rise to rickets. If Metchnikoff's views are correct, Pasteurization, which destroys the lactic acid bacilli but leaves many others of the *subtilis* group to multiply, is quite an irrational procedure.

This concludes that portion of the book which is directly concerned with the prolongation of life.

The next two parts are curiously disconnected from the problem of the prolongation of life. The first deals with the evidences of evolution displayed by the rudimentary organs in man and animals ; it attempts to explain certain psychic phenomena, such as somnambulism and hysteria, as reversions to ancestral states of mind, functional recrudescence, as it were, of conditions normally present, but in a rudimentary and latent way. The accounts of somnambulistic patients are curious and interesting, Then Professor Metchnikoff goes on to deal with "Some points in the History of Social Animals," his main thesis, which he supports by his usual wealth of biological detail, being that as the scale is ascended it becomes increasingly impossible to sacrifice the individual to the community; thus all theories of a socialist or collectivist character are essentially at fault, because they do not sufficiently regard the biological point of view—development of the individual, and secondarily of the State or rather of executive power. Metchnikoff, apparently looks to intellectual development to produce a better order of things in human society.

Thirty pages form Part VII., and are devoted to a survey of the Professor's favourite topics of optimism and pessimism. His view, it must be confessed, seem to us to be supported by somewhat fragmentary evidence. The poets and philosophers adduced as examples of the pessimistic frame of mind cannot be held to exert any preponderating influence on the majority of civilised mankind, with the sole exception of Buddha, who is claimed by the Professor as one of them. Byron, for instance, has almost vanished as an "influence," at any rate among his own countrymen. The curious life history of the reclaimed pessimist will be read with interest, and doubtless many will note that on a previous page (p. 130) the author states that he has twice experienced rather severe morphia poisoning himself. Towards the end of this part the view is propounded that pessimism in youth is succeeded by optimism in old age, and while this in a modified degree is often true, we cannot feel that it is anything like an accurate generalisation, at any rate for Anglo-Saxon peoples.

Finally, the relation between science and morality is discussed. Here is, to our mind, some of the most remarkable writing in the book. The admirable restraint and the fairness with which opposing views are stated is most remarkable, coming as it does from a man whose own opinions are evidently clearly formed and firmly held. His object is to find a basis of morality apart from any metaphysical sanction; and though we admire the way in which the subject is handled, it appears to us that the author has set himself an absolutely impossible task, and that his theory of orthobiosis is no more stable a foundation for a system of ethics than any of those the inadequacy of which he exposes. There are many eloquent passages in this portion of the book, but we venture to think that the substance of much of it has been expressed before, and that in point of view of eloquence the words of one whom he regards as a pessimist are more telling even than those of Professor Metchnikoff: "Happy is the man that findeth wisdom and the man that getteth understanding . . . length of days is in her right hand, and in her left hand riches and honour. Her ways are the ways of pleasantness and all her paths are peace."

